# The critical roles and therapeutic implications of tuft cells in cancer

**DOI:** 10.3389/fphar.2022.1047188

**Published:** 2022-12-09

**Authors:** Lin Li, Mengmeng Ma, Ting Duan, Xinbing Sui

**Affiliations:** ^1^ School of Pharmacy, Hangzhou Normal University, Hangzhou, China; ^2^ Department of Medical Oncology, The Affiliated Hospital of Hangzhou Normal University, Hangzhou Normal University, Hangzhou, China

**Keywords:** immune function, tuft cells, cancer therapy, POU2F3, DCLK1

## Abstract

Tuft cells are solitary chemosensory epithelial cells with microvilli at the top, which are found in hollow organs such as the gastrointestinal tract, pancreas, and lungs. Recently, an increasing number of studies have revealed the chemotactic abilities and immune function of the tuft cells, and numerous efforts have been devoted to uncovering the role of tuft cells in tumors. Notably, accumulating evidence has shown that the specific genes (POU2F3, DCLK1) expressed in tuft cells are involved in vital processes related with carcinogenesis and cancer development. However, the interaction between the tuft cells and cancer remains to be further elucidated. Here, based on an introduction of biological functions and specific markers of the tuft cells, we have summarized the functional roles and potential therapeutic implications of tuft cells in cancers, including pancreatic cancer, lung cancer, gastric cancer, colon cancer, and liver cancer, which is in the hope of inspiring the future research in validating tuft cells as novel strategies for cancer therapies.

## Introduction

Tuft cells are unusual epithelial cells that were firstly observed in an apical brush border of the rat trachea by Rhodin et al. 60 years ago (Rhodin and Dalhamn, 1956). And Jarvi et al. also reported the discovery of cells with similar morphological features in the mouse stomach in the same year (Jarvi and Keyrilainen, 1956). Later, the presence of tuft cells were detected in the human trachea (Rhodin, 1959). Over the past decades, transmission electron microscopy, scanning electron microscopy, and histochemical techniques have contributed to characterizing the morphological criterion for identifying these unusual epithelial cells, and “tuft” was finally used to describe the cellular morphology in detail (Isomaki, 1973; Luciano and Reale, 1979). In some circumstances, tuft cells have been referred to as “brush-like” “vesicular”, “peculiar”, “fibrillovesicular”, or “caveolated” cells, which all displayed the similar ultrastructures with a microvillus tuft (Reid et al., 2005; Hoover et al., 2017; Hendel et al., 2022). In this paper, we will refer to these cells collectively as tuft cells since they appear to be rather closely associated across tissues.

Generally, tuft cells are located in epithelium that are predominantly present at mucosal surfaces of vertebrates derived from endoderm. In the respiratory tract, tuft cells are distributed in the respiratory and olfactory epithelium of the nose, trachea, and proximal airway (O'Leary et al., 2022; Ualiyeva et al., 2020; Zaragosi et al., 2020). In the gastrointestinal tract, tuft cells are present in the stomach, small and large intestines, as well as the pancreatic bile tract (Hoover et al., 2017; O'Leary et al., 2019). Moreover, tuft cells are found in the columnar epithelium of the urethra, ear canal, conjunctival nasal cavity, the gallbladder bile ducts, and thymus (O'Leary et al., 2019; Bornstein et al., 2018; Miller et al., 2018; Minton, 2022).

Over the past decade, plenty of studies have shown the relationship between tuft cells and the biological processes closely related with cancer, which suggested the potential role of tuft cells in clinical applications of cancer treatment (Yamada et al., 2021; Yamada et al., 2022). Meanwhile, evidence from specific genes of tuft cells has also confirmed that the involvement of tuft cells in cancer. Doublecortin-like kinase1 (DCLK1), a characteristic gene of tuft cells, was associated with progression of gastric cancer, pancreatic cancer, colon cancer, and renal cancer (Weygant et al., 2016; Ge et al., 2018), and involved in the initiation, growth, metastasis, epithelial-mesenchymal transition (EMT), and stemness of the tumor (Westphalen et al., 2017; Sureban et al., 2011; Ito et al., 2016). Interleukin-25 (IL-25), one of the most important effector factors secreted by tuft cells, also plays an irreplaceable function in the immune processes (Hayakawa and Wang, 2018).

### Physiological functions of tuft cells

The entire gastrointestinal tract (the esophagus, stomach, and intestines) serves as a physical barrier and the largest immune organ against the outside environment, and is responsible for nutrient absorption and immune surveillance (Takiishi et al., 2017). Tuft cells are particularly useful in regulation of immune responses, especially for recognizing helminth and protist infection (Hendel et al., 2022). What’s more, as remote chemosensory epithelial cells, tuft cells facilitate the transmission of chemical signals *via* producing effector molecules such as IL-25, cysteinyl leukotrienes, DCLK1, Tas2r, etc. (Luo et al., 2019; Cao et al., 2020; Ualiyeva et al., 2021).

**FIGURE 1:**
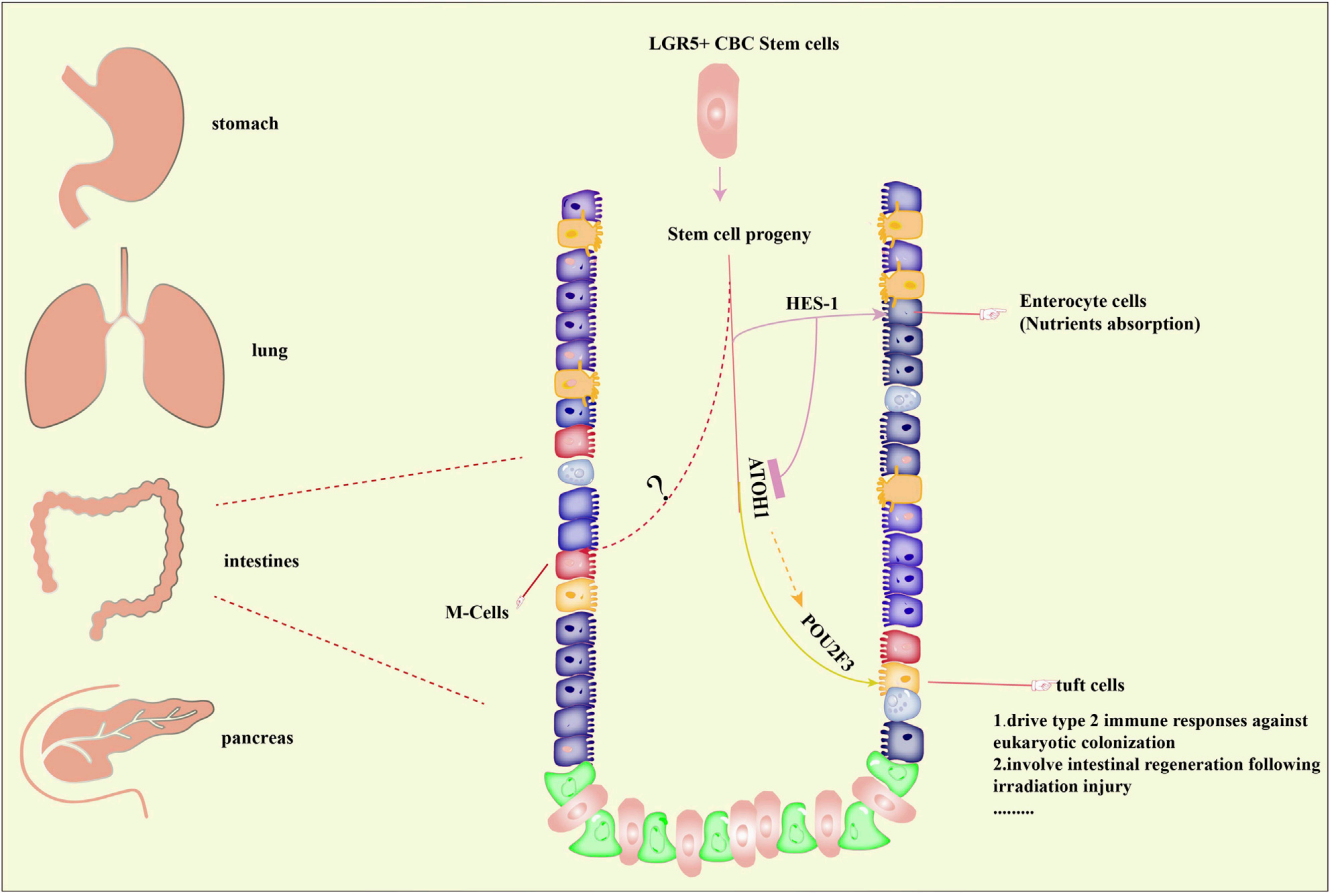
Distribution and formation of tuft cells. Tuft cells have been reported to be present in organs including the stomach, lung, intestine, pancreas. The formation of tuft cells in intestines has been predominantly studied and well elucidated. Briefly, stem cell progenitors originated from Lgr5+ crypt base columnar (CBC) stem cells can differentiate into tuft cells under the control of atonal homologue 1 (ATOH1) and POU Class 2 Homeobox 3 (POU2F3), both of which are master regulators of tuft cells. On the other hand, hairy/enhancer of split 1 (HES1) directs the formation of enterocyte cells, which is important for nutrients absorption. However, the underlying mechanisms of M-cells formation remains unclear. Importantly, tuft cells are associated with type 2 immune responses and regeneration of intestines.

As mentioned above, the presence of tuft cells was found in the lung, pancreas, stomach, intestine, and trachea. Significantly, the studies of tuft cells that focus on the intestine are relatively elaborated. Evidence from intestinal epithelial cell lines suggested that tuft cells originated from leucine-rich-repeat-containing G-protein-coupled receptor 5 (Lgr5^+^) stem cells were the source of tuft cells (van Es et al., 2019; McKernan and Egan, 2015). By using a knock-in mice model, the *in vivo* lineage tracing experiments demonstrated that Lgr5 served as a stem cell marker of tuft cells in the small intestine and colon (Barker et al., 2007). And hairy/enhancer of split 1 (HES1) or atonal homologue 1 (ATOH1) determines the cell fate into absorptive or secretory cell (Gerbe et al., 2011). In addition, POU2F3 is also crucial. The mechanism of M-cells formation deserves further exploration (Figure 1).

**FIGURE 2:**
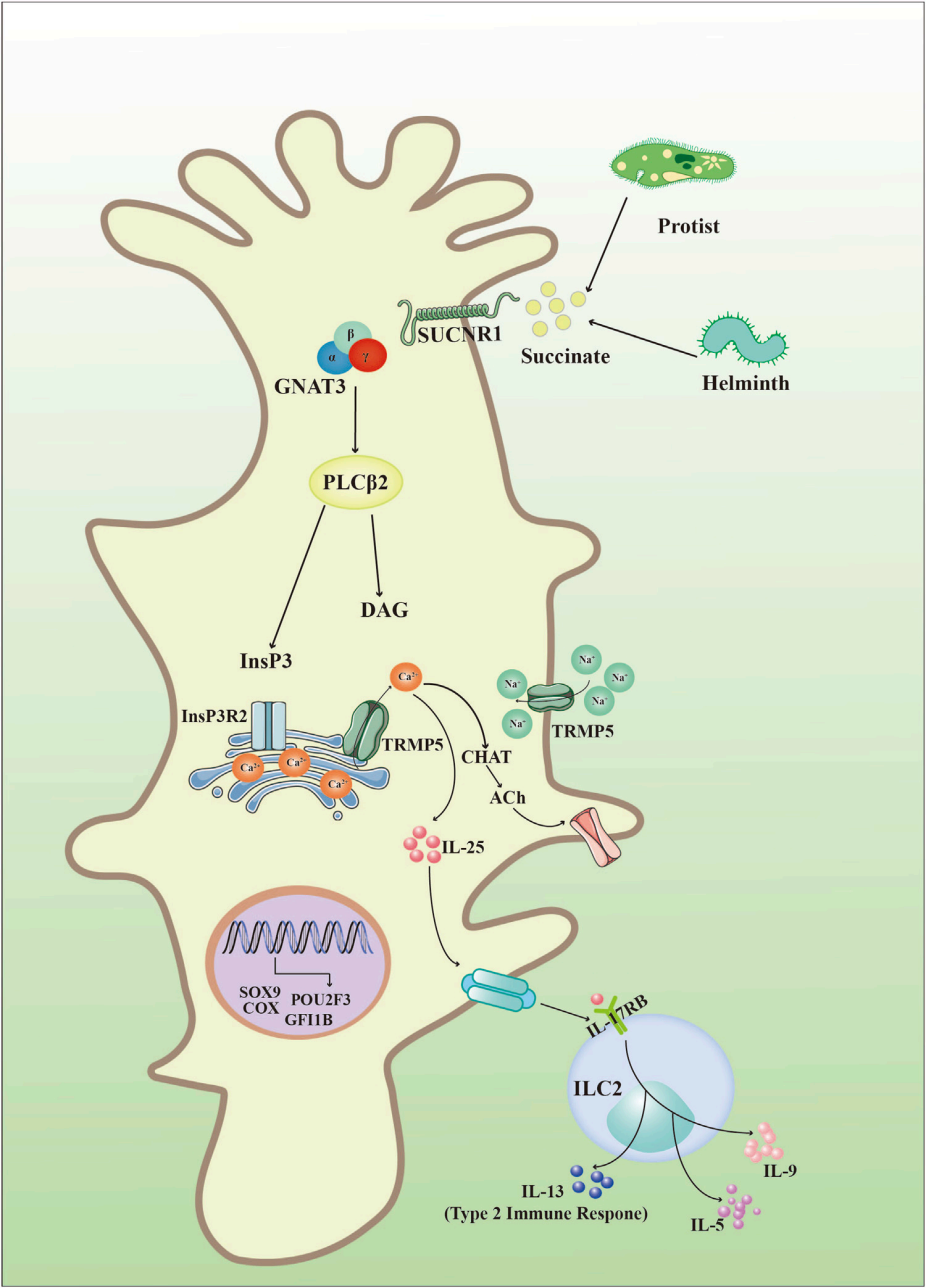
Intestinal tuft cell-specific signaling. Invasive organisms including helminths and protozoa produce succinate to bind with succinate receptor 1 (SUCNR1), which further interacts with guanine nucleotide-binding protein G(t) subunit α3 (GNAT3) and activates phospholipase Cβ2 (PLCβ2) to produce inositol-1,4,5- trisphosphate (InsP3) and diacylglycerol (DAG). InsP3 then binds to its receptors (InsP3Rs) in the endoplasmic reticulum and causes the release of Ca^2+^ into the cytoplasm. Subsequently, increased Ca^2+^ in the cytoplasm activates transient receptor potential cation channel subfamily M member 5 (TRPM5) and results in: (1) choline acetyltransferase (CHAT)-mediated release of acetylcholine (ACh); (2) secretion of IL-25 to bind with interleukin 17 receptor B (IL-17RB) in group 2 innate lymphoid cells (ILC2s) and synthesize cytokines such as IL-5, IL-9, and IL-13.

Intestinal helminths constitute a diverse group of pathogens which lead to infection of intestines in over one billion people world wide (Bąska and Norbury, 2022). To eliminate gastro-intestinal helminths, an integrated response orchestrated by epithelial, neural, and immune cells are used to expel the parasites, increased goblet cell mucus production and muscle contractions result in helminths expulsion from the gut and release outside the body, which is known as “weep and sweep” (Hildersley et al., 2021). Upon infection with helminths or protists, epithelial tuft cells release IL-25 to activate ILC2, which initiates type 2 immune response (von Moltke et al., 2016). Firstly, succinate receptor 1 (SUCNR1; also known as G protein-coupled receptor), a specific marker of tuft cells in small intestine and trachea (Nadjsombati et al., 2018), binds with succinate secreted by pathogens and leads to depolymerization of guanine nucleotide-binding protein G(t) subunit α3 (GNAT3) when the organism was infected with pathogens such as helminths (Schneider et al., 2019; Hoffman et al., 2021). Subunit βγ from GNAT3 activates downstream phospholipase Cβ2 (PLCβ2) to regulate cleavage of phosphatidylinositol-4,5-bisphosphate PtdIns (4,5) P2 (PtdIns (4,5) P2) into inositol-1,4,5- trisphosphate (InsP3) and diacylglycerol (DAG) (Rössler et al., 1998; Schneider et al., 2019). Then, InsP3 binds with its receptor (InsP3Rs) on the endoplasmic reticulum and causes the release of intracellular calcium (Ca^2+^) (Simon et al., 2006). Increased Ca^2+^ in the cytoplasm activates transient receptor potential cation channel subfamily M member 5 (TRPM5) inside tuft cells and leads to the flow of Na^+^ into tuft cells, which depolarizes the tuft cells (Liu and Liman, 2003), and elevated Ca^2+^ leads to choline acetyltransferase (CHAT)-mediated release of acetylcholine (ACh) (Fu et al., 2018; Hollenhorst et al., 2020). Importantly, ACh generated by tuft cells is involved in a variety of pathophysiological processes including epithelial homeostasis (Middelhoff et al., 2020; Westphalen et al., 2014), airway remodeling (Pieper et al., 2007), respiratory reflexes (Krasteva et al., 2011), inflammation (Ualiyeva et al., 2020), carcinogenesis (Huang et al., 2018), and ATP production (Dando and Roper, 2012). In addition, activated TRPM5 also promotes the secretion of IL-25 in tuft cells, which is an essential cytokine involved in type 2 immune responses (Nadjsombati et al., 2018; Harris, 2016). Epithelial tuft cells are the only source of IL-25, when IL-25 is released outside of tuft cells, it binds to receptors of innate lymphoid cells (ILC2) to promote the secretion of immune factors including IL-5, IL-9, and IL-13, then IL-13 promotes the formation of tuft cells and subsequently increases IL-25, which is regarded as a response circuit for type 2 immune responses in tuft cells (von Moltke et al., 2016; Howitt et al., 2016) (Figure 2). The researchers further identified and characterized a tuft-cell deficient mice line. The analysis found that POU2F3, DCLK1, and SOX9 were all absent, demonstrating the absence of tuft cells. The authors revealed the critical function of tuft-cell in initiating mucosal type 2 responses after infecting worms with IL-25 secretion. In the absence of tuft cells, IL-25 and IL-13 expression remained low, and type 2 mucosal reactions and helminth expulsion were delayed. In addition, tuft cells are required upstream of IL-4/IL-13. These cytokines drive tuft cells proliferation and thus amplify the feedforward loop to coordinate rapid and effective anthelmintic immunity (Gerbe et al., 2016; Schneider et al., 2018).

By producing cytokines such as IL-25 and ACh in various tissues, tuft cells show impacts on many physiological and pathological processes, which extends the understanding of the applications of tuft cells in diseases.

### Markers of tuft cells

It has been reported that tuft cells constitute the fifth cell type in the intestinal epithelium, thus, it is important to define tuft cells with specific characteristics. Although morphological features help to distinguish tuft cells from other chemosensory epithelial cells, the unique molecular characteristics of tuft cells are essential for the definition of tuft cells and the validation of their functions (Table 1).

**TABLE 1:** The markers of tuft cells.

Markers	Signatures	References
AcTub	Co-expressed with DCKL1 and TRPM5 in the adult gastrointestinal tract	Saqui-Salces et al. (2011); Ting and von Moltke, (2019)
Advillin	Expressed in tuft cells in the gastrointestinal tract, and involved in chemosensory signals and type 2 immune responses	Esmaeilniakooshkghazi et al. (2020)
ATOH1	Determines the formation of tuft cells from stem cells	Shroyer et al. (2007); Banerjee et al. (2020)
CHAT	An enzyme expressed by cholinergic tuft cells and mediates the transmission of acetylcholine	Schutz et al. (2015); Huang et al. (2022)
CK18	Co-localizes with villin in tuft cells and disperses around the nucleus and in the cytoplasm of tuft cells	Panneck et al. (2014); Nevo et al. (2019); (Delgiorno et al., 2014)
COX1 and COX2	Specifically expressed in healthy intestinal epithelium showing effects on prostaglandin biosynthesis	Adelizzi (1999), Wang et al., (2010)
DCLK1	A serine/threonine protein kinase that is associated with microtubules and is a marker of intestinal tuft cells	Westphalen et al. (2017); Gerbe et al. (2009)
GFI1B	A transcriptional repressor is constitutively expressed in all tuft cells	Bjerknes et al. (2012)
GNAT3	A G protein that is specifically expressed in normal tuft cells	Hoffman et al. (2021)
IL-25	Constitutively expressed in all tuft cells to mediate type 2 immune response of the small intestine	von Moltke et al. (2016); Gerbe et al. (2016); Howitt et al. (2016)
PLCβ2	Receives signals from upstream GNAT3 and mediates cleavage of PtdIns (4,5) P2	(Liu et al., 2003)
POU2F3	A transcription factor dominates the formation of tuft cells	Bornstein et al. (2018)
SOX9	It is associated with POU2F3 and TRPM5 in tuft cells	Yuan et al. (2021)
SUCNR1/GPR91	Most highly expressed in tuft cells of the small intestine	Lei et al. (2018); Nadjsombati et al. (2018)
TRPM5	A calcium-activated cation channel is expressed in tuft cells of the gastrointestinal tract and the respiratory system	Kaske et al. (2007); Howitt et al. (2016); McGinty et al. (2020)
TSLP	An epithelial cytokine is abundantly expressed in tuft cells in the small intestine	Schneider et al. (2019)

Pou class 2 homeobox 3 (POU2F3), also known as OCT-11 and Skn-1a (Huang et al., 2018), is constitutively expressed in all tuft cells and a specific master regulator for differentiation of tuft cells (Ohmoto et al., 2013), and POU2F3 deficiency results in the entire absence of tuft cells in mice (Gerbe et al., 2016). Although the underlying transcriptional mechanisms of POU2F3 in regulating formation of tuft cells remains poorly understood, it has been confirmed the binding of POU2F3 to POU2AF2 (also known as C11orf53 or OCA-T1) and POU2AF3 (also known as COLCA2 or OCA-T2) is important in the tuft cell lineage (Szczepanski et al., 2022; Wu et al., 2022). By using a Balb/cJ (Balb) mice strain with fewer intestinal tuft cells than C57BL/6J (B6) mice, it was identified that POU2AF2 isoform usage was a novel regulator of tuft cells differentiation and tuned innate type 2 immunity in the small intestine (Nadjsombati et al., 2022). In addition, POU2AF2 and POU2AF3 were identified as novel vulnerabilities in tuft cell-like SCLC and POU2AF3 functioned as a co-activator for POU2F3 to drive the transcriptional program critical for tuft cell-like SCLC (Zhou et al., 2022). It was found that 11q23.1 trans-eQTL (expression Quantitative Trait Loci) targets comprised a POU2AF2-related network, which might be specific for tuft cells and associated with abundance of tuft cells (Harris et al., 2022). Furthermore, by using a POU2F3 deficient mice strain Yamashita et al. demonstrated that POU2F3 as a master regulator for the generation of TRPM5-expressing chemosensory cells, like intestinal tuft cells, and was required for generation of tuft cells (Yamashita et al., 2017). ATOH1, also known as Math1, is another transcription factor that dominates the differentiation of tuft cells (Gerbe et al., 2011). It has been found that ATOH1 determined the fate of early differentiation of epithelial stem cells, which distinguishes tuft cells from other cell types of the intestinal epithelium including cup cells, discoid cells, and intestinal endocrine cells (Gerbe et al., 2011; Gracz et al., 2018). Notably, tuft cells were not present in ATOH1 knockout mice and differentiation of all secretory cells was impaired without ATOH1 (Shroyer et al., 2007). Thus, POU2F3 and ATOH1 are specific transcription factors in defining tuft cells, but more studies are needed to further illustrate the transcriptional program in regulating formation of tuft cells. SOX9, a member of the SOX gene family that regulates the development, cell-fate determination, and differentiation of multiple organs (Pritchett et al., 2011), has been confirmed as a marker of tuft cells (McKinley et al., 2017) and played a key role in tumor development and progression, such as glioma and colorectal cancer (Lü et al., 2008; Swartling et al., 2009). Studies have found that the expression of SOX9 was positively associated with transcription factors POU2F3 and TRPM5 (Yuan et al., 2021).

Besides those markers mentioned above, several other proteins involved in the regulatory function of the tuft cells have also been used to identify tuft cells. For example, DCLK1, a serine/threonine protein kinase associated with microtubules, is the most commonly used and prevalent marker in tuft cells (Westphalen et al., 2017; Yi et al., 2019). Arachidonate 5-lipoxygenase (ALOX5), an important enzyme for the synthesis of leukotrienes from arachidonic acid, is also observed extensively expressed in tuft cells (McGinty et al., 2020). Both cyclooxygenases 1 (COX1) and cyclooxygenases 2 (COX2) are key enzymes needed for the production of prostaglandin H2 (PGH2), and studies have confirmed that tuft cells were the only epithelial cells in the uninflamed intestine that expressed COX1 and COX2 (Sauvé, 2019; Zhang, 2021). Thus, the expression of COX1 and COX2 indicates the presence of tuft cells in the intestinal epithelium. Advillin, a member of the villin/gelsolin superfamily, is a sensory neuron-specific actin-binding protein (Marks et al., 1998) and has been identified as a specific marker of tuft cells in the epithelium of the gastrointestinal and biliary tract (Ruppert et al., 2020). Studies have shown that advillin binds to F-actin and regulates the migration of cancer cells (Xie et al., 2020). In addition, advillin is responsible for the depolymerization of gelsolin and villin (Khurana, 2006). Although the function of advillin has not been fully studied, it was reported to be involved in the inflammatory response to helminths and bacterial microbiota cells and participated in the transmission of sensory signals to the enteric nervous system as well as type 2 immune responses (Esmaeilniakooshkghazi et al., 2020). In addition, proteins including SUCNR1, TRPM5, CHAT, and IL-25 that are involved in intestinal tuft cell-specific signaling are also regarded as critical markers of tuft cells.

Accumulative advances have gradually uncovered the significance of tuft cells in human diseases. Although increasing cell markers are being discovered and their association with diseases has been identified, much more efforts should be devoted to elucidating the underlying mechanisms and the potential of tuft cells in diseases including cancer.

### The potential roles and underlying mechanisms of tuft cells in cancer

Over the past decade, increasing evidence from genes specifically expressed in tuft cells has proven the significance of tuft cells in the development and progression of cancers including pancreatic cancer, lung cancer, and colon cancer. Here, we will summarize the functions and underlying mechanisms of tuft cells in those tumors.

### Pancreatic cancer

It has been reported that two-thirds of neuroendocrine carcinoma (NEC) were found present in the digestive system, and the pancreas cancer is among the most common ones (Yachida et al., 2022). Pancreatic cancer is one of the prevalent malignancies of the digestive tract and is regarded as the king of carcinoma (Luo et al., 2020). Tuft cells have been found in both human and mouse pancreas, and play a significant role in pancreatic cancer (Schütz et al., 2019; DelGiorno et al., 2020a).

By using mouse models, a massive increase of tuft cells was found during chronic pancreatitis and acinar-to-ductal metaplasia (ADM) in response to pancreatic injury, and tuft cells were transformed from alveolar cells following complex changes, which was similar to the process that a reservist (alveolar cells) leaped from a civilian to a warrior (tuft cells) to fight against inflammatory factors (DelGiorno et al., 2020b). In a KC mice model of pancreatic ductal adenocarcinoma (PDAC), increased in tuft cells and elevated prostaglandin D2 (PGD2) levels were found in KC mice in comparison with that in wild-type mice. In addition, deficiency in the differentiation of tuft cells by silencing POU2F3 resulted in decreased tuft cells and inhibition of the progression of PDAC in KC mice (DelGiorno et al., 2020a). However, in LSL-KRAS^G12D/+^ and Ptf1a^Cre/+^ mice models of pancreatic tumorigenesis, researchers found that tuft cells were most abundant in ADM and became less abundant during disease progression, with 11.2% of mPanIN-1 epithelium, 8.7% of mPanIN-2, and 2.9% of mPanIN-3, while tuft cells were absent in invasive disease. In addition, they also found that abnormality of pancreatic tuft cells was common in KRAS^G12D^-induced pancreatic disease and is accompanied by an epithelial expression with SOX17, which promoted KRAS^G12D^-induced tumorigenesis in mice (Delgiorno et al., 2014). It was also found that overexpression of IL-17RB was associated with metastasis and poor clinical outcome of pancreatic cancer, and impairing IL-17B-IL-17RB signaling blocked metastasis of pancreatic cancer (Wu et al., 2015).

### Lung cancer

Tuft cells are scattered as isolated cells in the epithelium of the airways and are generally absent in the alveoli except in cases of post-viral remodeling (Rane et al., 2019). In 2018, a small cell lung cancer (SCLC) derived from tuft cells was identified, and a tuft cell-like gene expression profile was found in this type of SCLC, which was characterized by exclusively expression of POU2F3 (Huang et al., 2018). Tuft cell-like tumors with co-expression of POU2F3, GFI1B, and KIT were identified among pulmonary squamous cell carcinomas, adenocarcinomas, and large cell neuroendocrine carcinoma, and new molecular subsets were delineated by their tuft cell-like signatures among the lung cancer histotypes (Yamada et al., 2021).

Pathologically, POU2F3-positive tumors were defined as one type of true small cell lung cancer with specific morphological characteristics and extremely high proliferation rates (Baine et al., 2020). Based on the critical role of POU2F3 in lung cancer, it was uncovered that binding between POU2F3 and its coactivators (OCA-T1, OCA-T2) was essential in tuft-cell-like small-cell lung cancer, and POU2F3–OCA-T complex was the master regulator for identity of tuft cells and a potential target in treating tuft-cell-like small-cell lung cancer (Wu et al., 2022). Thus, the therapeutic strategy that aims at blocking POU2F3 function or ablating the tuft cell lineage is expected to have a wide therapeutic scope for patients with tuft cell-like lung cancers.

Considering the extremely poor prognosis of tuft-cell-like small-cell lung cancer, it is urgent to improve the early diagnosis and treatment options for those patients. Specific markers of tuft cells such as POU2F3 may be an Achilles’ heel for tuft cell-like lung cancer patients, but much more is needed to further clarify the relationship of tuft cells with lung cancer.

### Gastric cancer

Emerging evidence has been suggesting the oncogenic function of tuft cells in gastric cancer. High expression of POU2F3 was found in three neuroendocrine carcinoma of the gastrointestinal systemincluding colon, esophageal and gastric cancers (Yachida et al., 2022). Inhibition of the tuft cell-ILC2 axis by genetic ablation of tuft cells, ILC2s or antibody-mediated neutralization of IL-13 or IL-25 attenuated gastric tumor development in mice (O’Keefe et al., 2022). DCLK1-expressing tuft cells are one of the two main sources of ACh within the gastric mucosa (Hayakawa et al., 2017). Furthermore, dual immunofluorescence analysis of cholinergic marker CHAT and tuft cells specific markers such as advillin identified that tuft cells were the unique source for epithelial biosynthesis of ACh in the human alimentary tract (Schütz et al., 2019). Notably, it was found that ACh secreted by DCLK1^+^ tuft cells and nerves within the gastric mucosa stimulated nerve growth factor (NGF) expression in gastric epithelium to promote gastric carcinogenesis, and ablation of DCLK1^+^ cells inhibited epithelial proliferation and tumorigenesis (Hayakawa et al., 2017). Thus, this feed-forward ACh-NGF axis derived by tuft cells was recommended as a compelling target for the prevention and treatment of gastric carcinoma. In conclusion, tuft cells play an important role in gastrointestinal tumors.

### Colon cancer

In the small intestine, tuft cells are rarely present under homeostasis, but infection with various related pathogens will promote the differentiation of Lgr5 stem cells into tuft cells, and ultimately increase the number of tuft cells and goblet cell fraction to promote pathogen clearance (von Moltke et al., 2016). In the case of tissue damage, intestinal progenitor cells would proliferate quickly and differentiate into tuft cells to promote type 2 immunity to eliminate inflammation and prevent the progression of ulcerative colitis (Sunaga et al., 2022). In addition, berberine, the main active component of an ancient Chinese herb *Coptis Chinensis French*, was found to regulate the repairment of damaged intestinal tract by promoting the IL-25-ILC2-IL-13 immune pathway in tuft cells (Xiong et al., 2021). Regarding that ulcerative colitis is usually considered to be a precancerous lesion of colon cancer (Kvorjak et al., 2020), it is believed that tuft cells may be involved in the initiation of colon cancer.

By using a lineage-tracing assay, it was found that DCLK1 marked tumor stem cells that continuously produce tumor progeny rather than normal stem cells in the intestine, and specific ablation of DCLK1-positive tumor stem cells lead to significant regression of polyps without significant damage to the normal intestine (Nakanishi et al., 2013). It has been reported that DCLK1 played an important role in promoting intestinal tumorigenesis, and depletion of DCLK1 reduced the stemness and inhibited progression of tumors, thus suggesting its role in regulating pro-survival signals and tumor cell pluripotency (Chandrakesan et al., 2017). Thus, tuft cells may serve as a novel choice in the treatment of colon cancer by targeting DCLK1-positive cancer stem cells. Similarly, IL-17RB expression was also found in human colorectal cancer stem cells instead of normal stem cells by genealogical tracking, and long-term ablation of IL-17RB resulted in the inhibition of cancer stem cells growth *in vivo*, which all validated the implication of IL-17RB^+^ cancer stem cells as clinical targets for cancer therapy (Goto et al., 2019). Moreover, overexpression of SOX9 has been shown to be involved in human colorectal carcinogenesis by *in vitro* and *in vivo* studies (Reineking et al., 2022).

### Liver cancer

The presence of tuft cells in the liver has not been confirmed yet. However, it was found hyperplastic epithelial tuft cells in the colon induced by gut microbiota dysbiosis might partly be the source of IL-25 and increased IL-25 promoted the progression of hepatocellular carcinoma (HCC) *via* alternative activation of macrophages in the tumor microenvironment (Li et al., 2019). Thus, it is hypothesized that colonic epithelial tuft cells could promote HCC progression by secretion of effector factors.

In summary, we have summarized the role of tuft cells in tumors and indicated the potential possibilities of tuft cells as a novel target for tumor treatment, and strategies targeting specific molecules including DCLK1 and POU2F3 can become good choices for cancers with tuft cell signatures. Therapeutic approaches targeting specific markers of tuft cells by kinase inhibitors, monoclonal antibodies, and chimeric antigen receptor T cells (CAR-T) are preferred (Cao et al., 2020). However, much more remains to be explored in deepening our understanding of tuft cells.

### Heterogeneity of tuft cells

As rare chemosensory epithelial cells and critical orchestrators in mediating type-2 immunity (Gerbe et al., 2012), tuft cells have been revealed to be heterogeneous in their lineage and functions. Recent advances from single cell sequencing confirmed diversity of individual tuft cells (Haber et al., 2017). By using single-cell survey of small intestinal epithelial cells, tuft cells were clustered into four subsets including two representing progenitors (early and late) and two representing mature cells (tuft-1 and tuft-2) (Haber et al., 2017). In response to helminth infections, protozoan colonization, and bacterial ecological dysregulation in the small intestine, tuft cells initiate type 2 immune responses characterized by tissue remodeling. In the airway, tuft cells sense external noxious stimuli through their own functions and make a series of corresponding responses (Billipp et al., 2021). When infected with protist or when mice are treated with succinate to mimic protist infection, no tuft cell hyperplasia is observed in the gallbladder, colon, and cecum (von Moltke et al., 2016; Nadjsombati et al., 2018). And it has been reported that tuft cells were increased in the small intestine and DCLK1^+^ tuft cells were largely absent in the colon in the ATOH1 ablation mice model (Herring et al., 2018), which suggested that ATOH1 might be required for colonic tuft cells differentiation. Also, the proliferation of intestinal tuft cells is influenced by the luminal environment, and when invaded by microbiota, colonic tuft cells of germ-free mice are temporarily increased (McKinley et al., 2017). A20 (Tnfaip3) deficient mice (Tnfaip3loxP/loxP; IL5RFP-Cre) only have tuft cell hyperplasia in the small intestine but not the colon, cecum, and stomach (Schneider et al., 2018). In addition, tuft cells in the thymus promoted the canonical taste transduction pathway and secreted IL-25, which was similar with that in the intestine. On the other hand, thymic tuft cells were unique in their ability to present antigens (Miller et al., 2018). Thymic tuft cells also express a variety of typical taste receptors that have been described in airway tuft cells but seem to be largely absent in intestinal tuft cells (Krasteva et al., 2011; Nadjsombati et al., 2018), which suggested that tuft cells from different tissues have distinct cellular properties, which potentially can be traced back to distinct gene expression programs (Haber et al., 2017; Bornstein et al., 2018; Plasschaert et al., 2018; Shanahan et al., 2021). Even within the same tissue, there are different types of tuft cells. For example, in the intestine, tuft cells have been found to have two subtypes, which are termed tuft-1 and tuft-2. Tuft-2 cells are associated with immunity, while tuft-1 cells are associated with neuronal development (Haber et al., 2017). Tuft cells are also heterogeneous in injury-inducing lung models, Barr et al. have found transcriptional heterogeneity in this lung tuft cell, and the locus from 'basal- > tuft' to 'tuft-2' and 'tuft-1' clusters was identified. Although the tuft cells in the injured lung were consistent with intestinal tuft cells in their POU2F3 dependence, lung tuft cells were not dependent on type 2 immune signaling, unlike the intestinal (Barr et al., 2022). Different from the intestinal and the injury-inducing lung, in the airway, tuft cells are divided into three clusters, immature tuft, tuft-1, and tuft-2 cells. Tuft-1 expresses genes related to taste transduction, and tuft-2 is associated with immunity (Montoro et al., 2018).

## Discussion

Increasing studies have been focused on tuft cells, and the impacts of tuft cells are gradually being reported. As a relatively rare epithelial cell population, tuft cells exhibit a specific expression profile, and are involved in the processes of cell repair, inflammation, and parasite-induced type 2 immunity.

In recent years, the role of tufts cells in tumors has been increasingly reported and their role in tumors is gaining attention. In this review, based on the introduction of the physiological functions and specific markers of tuft cells, we have summarized the roles and underlying mechanisms of tuft cells in cancer, in hope of inspiring more research in this field and providing clues for scientists with interests in tuft cells. Here, we demonstrated that tuft cells were increased in pancreatic ductal adenocarcinoma, small cell lung cancer, and colon cancer, and ablation of tuft cells by silencing specific markers inhibited the progression of pancreatic ductal adenocarcinoma and colon cancer, which all indicated the tumor-promoter role of tuft cells in cancer. These unique gene markers of tuft cells also help to explore the association of tuft cells with diseases including cancer. For example, DCLK1 was found up-regulated in renal clear cell cancer and colorectal cancer (Weygant et al., 2015; Gao et al., 2016; Mohammadi et al., 2018). As a potential effector of KRAS, DCLK1 contributes functionally to the pathogenesis of pancreatic cancer, and marks quiescent pancreatic progenitor cells of pancreatic origin (Westphalen et al., 2016). In addition, DCLK1-IN-1, a highly selective inhibitor with good uptake and bioavailability properties that targets DCLK1, has been proven to inhibit tumorigenesis in DCLK1^+^ organs (Ferguson et al., 2020). However, the role of DCLK1 in tuft cells remains to be further elucidated. Thus, we hypothesized that the functions of tuft cells might vary in cancers depending on the tumor types and stages. Considering the critical role of tuft cells in type 2 immunity, it is also inferred that tuft cells could be potential targets in uncovering novel immunotherapy strategies. Tuft cells have shown great potential in the development of antitumor drugs, but more evidence is still needed to illustrate the functions and underlying mechanisms, which will hold the promise of clinical application in tumors by targeting tuft cells.

However, the limited number of tuft cells as chemoreceptors in tissues is one of the obstacles to expanding their implications in cancer treatment, and it remains to be clarified whether cooperation with other epithelial cells is required to fulfill the functions of tuft cells. And it is of great importance to reveal more specific genes in tuft cells to facilitate studies in this field. With a comprehensive understanding of tuft cells, we hope drugs targeting tuft cells will benefit patients with tuft cell-like cancers.
